# The impact of goal-directed fluid therapy on postoperative pulmonary complications in patients undergoing thoracic surgery: a systematic review and meta-analysis

**DOI:** 10.1186/s13019-024-02519-y

**Published:** 2024-02-05

**Authors:** Shuang Han, Xiaoqian Wu, Pan Li, Kun He, Jianli Li

**Affiliations:** 1 Department of Anesthesiology, Hebei General Hospital, No.348, Heping West Road, Shijiazhuang, 050051 China; 2 Department of Anesthesiology, The Fourth Hospital of Shijiazhuang, No.16, Tangu North Street, Shijiazhuang, 050051 China

**Keywords:** Goal-directed fluid therapy, Thoracic surgery, Pulmonary complications, Pneumonia, Meta-analysis

## Abstract

**Background:**

Pulmonary complications after thoracic surgery are common and associated with significant morbidity and high cost of care. Goal-directed fluid therapy (GDFT) could reduce the incidence of postoperative pulmonary complications (PPCs) and facilitate recovery in patients undergoing major abdominal surgery. However, whether GDFT could reduce the incidence of PPCs in patients undergoing thoracic surgery was unclear. The present meta-analysis was designed to assess the impact of Goal-directed Fluid Therapy on PPCs in patients undergoing thoracic surgery.

**Methods:**

Randomized controlled trials (RCTs) comparing GDFT with other conventional fluid management strategies in adult patients undergoing thoracic surgery were identified. Databases searched included PubMed, Web of Science, Embase, and Cochrane Library databases. Review Manager 5.4 (The Cochrane Collaboration, Oxford, UK) software was used for statistical analysis. Heterogeneity was analyzed using I^2^ statistics, and a standardized mean difference with 95% CI and *P* value was used to calculate the treatment effect for outcome variables. The primary study outcomes were the incidence of PPCs. Secondary outcomes were the total volume infused, the length of hospitalization, the incidence of cardiac complications, and the incidence of renal dysfunction. Subgroup analysis was planned to verify the definite role of GDFT.

**Results:**

A total of 6 RCTs consisting of 680 patients were included in this meta-analysis, which revealed that GDFT did not reduce the incidence of PPCs in patients undergoing thoracic surgery (RR, 0.57; 95% CI 0.29–1.14). However, GDFT decreased the total intra-operative fluid input (MD, − 244.40 ml; 95% CI − 397.06 to − 91.74). There was no statistical difference in the duration of hospitalization (MD; − 1.31, 95% CI − 3.00 to 0.38), incidence of renal dysfunction (RR, 0.62; 95% CI 0.29–1.35), and incidence of cardiac complications (RR, 0.62; 95% CI 0.27–1.40).

**Conclusions:**

The results of this meta-analysis indicate that GDFT did not reduce the postoperative incidence of pulmonary complications in individuals undergoing thoracic surgery. However, considering the small number of contributing studies, these results should be interpreted with caution.

**Supplementary Information:**

The online version contains supplementary material available at 10.1186/s13019-024-02519-y.

## Background

Postoperative pulmonary complications (PPCs) including atelectasis, pneumonia, respiratory failure, and exacerbation of underlying chronic lung disease, adversely influence surgical morbidity and mortality. As reported, the incidence of PPCs in major surgery ranges from < 1 to 23% [1]. A systematic review and meta-analysis showed that attributable mortality due to postoperative lung injury is higher after thoracic surgery than after abdominal surgery [2]. Therefore, it is critical to reduce the occurrence of PPCs in patients undergoing thoracic surgery.

There are many causes of PPCs, such as inflammation, extensive tissue destruction, and one-lung ventilation. Furthermore, excess fluid administration has been linked to the higher incidence of PPCs following thoracic surgery [3]. Indeed, such overcapacity could exert an excess burden on the cardiac and pulmonary systems, resulting in potential adverse events or outcomes, including pulmonary edema, pulmonary infection, and delayed recovery of intestinal function [4, 5]. Current research evidence indicated that restrictive fluid regimens were appropriate for individuals undergoing pulmonary resection surgery and esophagectomy [6, 7]. However, these fluid-restrictive regimens may lead to potential hypovolemia or tissue hypoperfusion due to insufficient infusion. Therefore, appropriate perioperative fluid management is a crucial clinical consideration for patients undergoing thoracic surgery to reduce PPCs.

In recent years, the strategies to reduce the incidence of PPCs after thoracic surgery include lung-protective ventilation and goal-directed fluid therapy (GDFT). GDFT based upon fluid responsiveness has emerged as a perioperative approaches to ensuring appropriate fluid administration [8–10]. GDFT could maintain hemodynamic indices to ensure the volume is within the normal range necessary for appropriate tissue and organ perfusion [11]. A meta-analysis suggested that GDFT using fluids with positive inotropes and vasopressors could reduce the development of postoperative pulmonary infections and pulmonary edema in general, abdominal and cardiothoracic surgical patients [12].

Several studies identified a reduction in the incidence of postoperative complications and the length of hospitalization among thoracic surgery patients undergoing GDFT [13, 14]. In contrast, others suggested that GDFT during oesophageal resection did not reduce the incidence of postoperative complications. [15]. Therefore, there is still controversy about whether GDFT can decrease PPCs. Based on the previous research, we hypothesized that GDFT could reduce the incidence of PPCs in individuals undergoing thoracic surgery.

## Objectives

Our objective is to establish whether perioperative GDFT could reduce the incidence of PPCs in individuals undergoing thoracic surgery. We conducted the present systematic review and meta-analysis of relevant studies published to date.

## Methods

The Cochrane Handbook for Systematic Reviews of Interventions [16] and the Preferred Reporting Items for Systematic Reviews and Meta-Analyses (PRISMA) guidelines were used to guide the design and reporting of the present meta-analysis [17], (Additional file 1: Prisma Checklist) The protocol of the study has been registered in PROSPERO (CRD42021258820).

### Inclusion and exclusion criteria

Studies eligible for inclusion in the present study were selected based on patient, intervention, comparison, outcome, and study design strategy criteria [17]:

1. *Patients* Eligible patients were adult (≥ 18 years old) individuals undergoing thoracic surgery (thoracoscopic lobectomy, via thoracotomy lung parenchyma resection, open transthoracic esophagectomy, or thoracolaparoscopic esophagectomy).
2. *Intervention* GDFT intervention type was defined as perioperative administration of fluids with or without positive inotropes/vasoactive drugs to increase blood flow based on defined metrics. Four categories of hemodynamic monitoring approaches were employed: non-invasive, minimally invasive, esophageal Doppler, and pulmonary artery catheter-based techniques. All invasive and non-invasive monitoring devices that utilized pre-defined algorithms to direct flow towards particular hemodynamic targets were considered forms of GDFT, with used metrics including cardiac output (CO), stroke volume (SV), cardiac index (CI), stroke volume variation (SVV) oxygen delivery (DO2), oxygen consumption (VO2). Included studies were those for which GDFT was conducted during the intraoperative period.
3. *Comparator type* As a control group in the present study, conventional fluid intervention strategies reliant upon standard monitoring parameters (BP, HR, urine output, and CVP) for guidance were included in this study.
4. *Outcome type* Outcomes included the incidence of pulmonary postoperative complications, including pneumonia, pulmonary edema, acute lung injury, pulmonary hyperemia, and pulmonary infection.
5. *Study types* All relevant randomized controlled trials (RCTs) with or without blinding that had been published in English were included in the present study. We excluded case reports, reviews, cohort studies, or letters from this analysis.

### Data sources and search strategy

Relevant studies were identified by searching the Embase, PubMed, Cochrane Library, and Web of Science databases for all relevant RCTs of thoracic surgery patients comparing outcomes associated with GDFT and conventional fluid therapy approaches published as of June 30, 2022, that included at least one clinical outcome of interest. Search terms included the following: Pneumonectomy (lung resection, lobectomy), esophagectomy (esophagectomy, esophagectomies, oesophagectomies), One-lung ventilation (OLV, single-lung ventilation), Early Goal-Directed Therapy (GDFT, goal-directed fluid therapy), Fluid Therapy (fluid restriction, fluid optimization, fluid administration). For further details regarding the PubMed search strategy, see the additional file (Additional file 2: Search details). References of identified articles and studies were also searched to identify other potentially relevant studies. Only English language studies were eligible for inclusion in this meta-analysis.

### Data extraction and analysis

Two investigators (SH and XQW) independently reviewed potentially relevant studies, extracted data, analyzed RCT quality, and assessed the results of all analyses. Full-length articles were retrieved and reviewed when abstract review was insufficient to establish study eligibility, and disagreements were resolved through consensus. Data were extracted from studies using a pre-designed standard form based upon recommendations from the Cochrane Anaesthesia Review Group, with discrepancies resolved through discussion and consensus. Extracted data included: first author, country, publication year, study characteristics (design, randomization strategy), research object, number of cases, general study object data, intraoperative fluid administration approach (i.e., GDFT, other optimization goals, monitoring devices, fluid management), and outcomes (i.e., PPCs, total volume infused, renal dysfunction, cardiac complications, duration of hospitalization). When specific data were unavailable, efforts were made to contact the original study's authors to obtain missing information. When this was not successful, studies were excluded from this meta-analysis.

### Risk-of-bias assessment

Two investigators (SH and XQW) independently assessed the risk of bias associated with included RCTs using the Cochrane Collaboration tool [16]. Disagreements were resolved through discussion and consensus. Bias risk was defined as being low, high, or unclear for the following factors: blinding approach, random allocation approach, hidden allocation scheme, selective outcome reporting, incomplete outcome data, and other sources of bias. The risk of bias was judged based on all reports derived from a given study and from the original published protocol when applicable.

### Outcomes

The primary study outcomes were the incidence of PPCs. Secondary outcomes were the total volume infused, the length of hospitalization, the incidence of cardiac complications, and the incidence of renal dysfunction.

### Statistical analysis

The RevMan 5.4.1 software (The Cochrane Collaboration, 2020) was used to conduct the present analyses. Dichotomous variables were assessed using risk ratios (RRs) with the Mantel–Haenszel method using random effects models with corresponding 95% confidence intervals, with differences between groups being considered significant when the 95% CI did not include 1.0. Continuous data were assessed based on weighted mean difference (WMD) values and 95% CIs, with differences between groups being considered not significant when 0 were included within the 95% CI. The I^2^ statistic was used to assess heterogeneity, which was considered to be significant when I^2^ > 50%, in which case data were analyzed with a random-effects model, whereas a fixed-effects model was otherwise used. Subgroup analyses were used to identify sources of clinical heterogeneity where appropriate based on surgery type and device type utilized to measure particular hemodynamic goals. Data were transformed when insufficient data was available to yield mean and standard deviation values from presented medians with ranges, interquartile ranges, 95% CIs, or percentiles as per the Cochrane Collaboration criteria. Multiple comparison correction was performed using Bonferroni correction. The certainty of evidence was evaluated using the GRADE approach. However, assessing some domains was anticipated to be challenging, and if formal assessment could not be adequately performed, this step was excluded from the final analysis. We made the GRADE (grading of recommendations, assessment, development, and evaluation) assessment with the web‐based tool GRADE Pro GDT and classified them as high, moderate, low, or very low, according to the GRADE system.

## Results

**Fig. 1:**
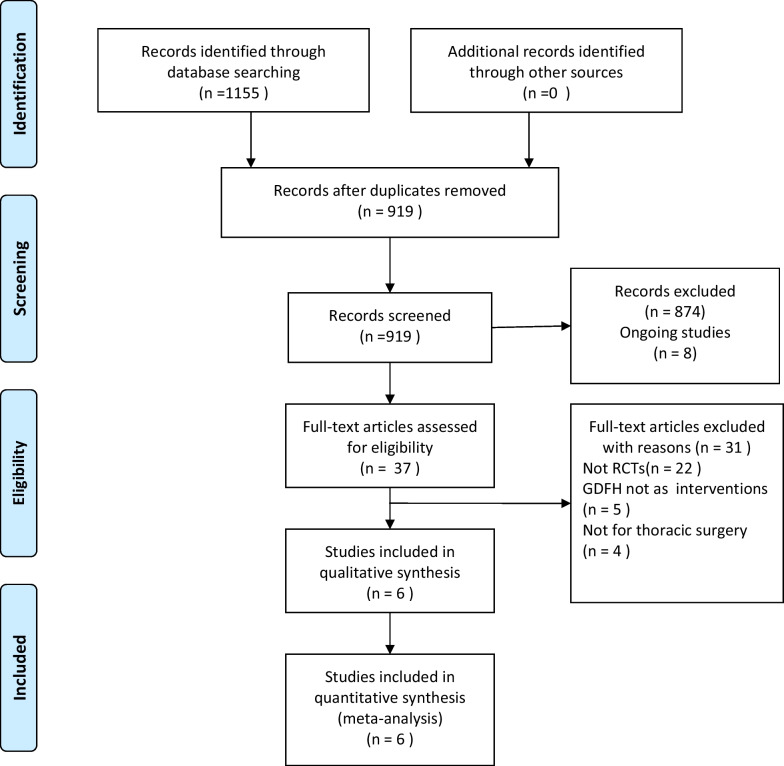
Preferred Reporting Items for Systematic Reviews and Meta-Analyses (PRISMA) diagram of study selection. GDFT Goal-directed fluid therapy, RCT Randomized controlled trial

An initial search yielded 1155 potentially relevant studies for title and abstract screening, of which 919 were excluded because they were not original studies, did not include human patients, or contained duplications of published data. The remaining 37 articles were subjected to further review based on relevant inclusion criteria. Of these articles, 31 were ultimately excluded as they were not RCTs, included non-surgical patients, did not assess the impact of GDFT, did not focus on thoracic surgery, did not include a conventional fluid therapy control group, or were published solely as abstracts or letters. The references of the remaining 6 articles, all of which were RCTs and considered eligible for study inclusion, were also searched for other potentially relevant studies. The PRISMA flow chart for the present study is shown in Fig. 1.

### Study characteristics

A total of 680 patients were included in the 6 RCTs [13–15, 18–20] included in the present analysis (Table 1). Of these patients, 340 underwent perioperative GDFT, with sample sizes ranging from 59 to 232 in individual studies. All of these articles were published in English language journals from 2013 to 2021.

**Table 1:** Included study characteris

Trial/author, year [reference]	Sample size	Male/female		Age (y)		Type of surgery	Timing	GDFT technology	Control group	Hemodynamic monitor	Interventions
		Control group	GDFT group	Control group	GDFT group						
Xu et al. [13]	168	56/28	54/30	49 ± 5	49 ± 6	Thoracoscopic lobectomy	Intraoperative	10% < SVV < 13% Cardiac index ≥ 2.5L/min/m^2^	Conventional fluid management according to the principles of Miller’s Anesthesia or used vasoactive substances if necessary	FloT rac/Vigileo device	Fluids, inotropes, and vasopressors
Bahlmann et al. [15]	59	21/8	24/6	66 ± 10	65 ± 7	Ivor Lewis oesophageal resection, McKeown oesophageal resection and left thoraco-abdominal approach	Intraoperative	SV optimisation, Cardiac index ≥ 2.5 L/min/m^2^ Mean arterial pressure (MAP) of at least 65 mmHg	The responsible anesthetist determined the fluid administration rate and use of vasoconstrictors and inotropes	A FloTrac pressure transducer Vigileo monitor	Fluids, inotropes, and vasopressors
Kaufmann et al. [14]	96	27/21	26/22	65 (55–74)	65 (56–70)	Lung parenchyma resection,via thoracotomy or video-assisted thoracoscopy	Intraoperative	SV optimisation, Cardiac index ≥ 2.5L/min/m^2^ Mean arterial pressure (MAP) of at least 70 mmHg	Conventional fluid and hemodynamic management according to standard operating procedures	Oesophageal Doppler guided	Fluids, inotropes, and vasopressors
Mukai et al. [19]	232	96	91	36 ± 82	42 ± 83	Open trans-thoracic oesophagectomy or thoraco-laparoscopic oesophagectomy	Intraoperative	SV optimisation, SVV < 12%, SBP > 90 mmHg	Intraoperative hemodynamic management sought to maintain a systolic BP > 90 mm Hg	Vigileo-FloTrac system	Fluids, inotropes, and vasopressors
Zhang et al. [18]	60	14/16	12/18	61.0 ± 8.7	59.9 ± 8.9	Thoracoscopic lobectomy	Intraoperative	GDFT technology	Control group	Vigileo-FloTrac system	Fluids, inotropes, and vasopressors
Wei Tang et al. [20]	65	23/9	28/5	70 ± 5	69 ± 3	Three-stage esophagectomy through the modified McKeown thoracoscopic, laparoscopic and cervical approach	Intraoperative	SVV optimization, SVV < 9%	Conventional fluid management	Pulse-contour analysis catheter (PiCCO™, pv2014L16-A, 4F)	Fluids, inotropes, and vasopressors

### Risk-of-bias assessment

**Fig. 2:**
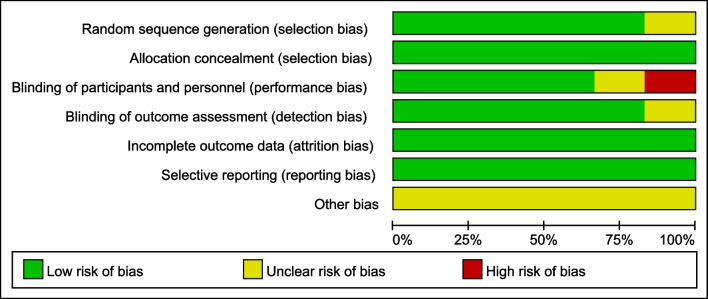
Risk of bias graph: Review authors’ judgements regarding each risk of bias item presented as percentages across all included studies. Green indicates no risk of bias, yellow and red represent unclear risk and high risk, respectively

The Cochrane tool was used to assess the risk of bias, showing the overall risk in Fig. 2.

Four of 6 studies were rated as exhibiting a low risk of bias [13, 15, 19, 20], 1 study exhibited a moderate risk of bias [14], and 1 study exhibited a high risk of bias [18].

### Publication bias

We did not use a funnel plot to assess publication bias owing to the low number of included studies in each analysis.

## Primary outcomes

### Pulmonary complications

**Fig. 3:**
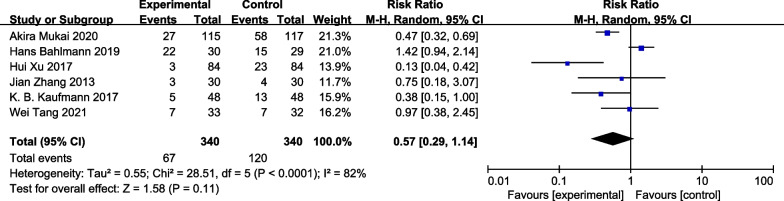
Meta-analysis and pooled risk ratio (RR) regarding the effect of perioperative goal-directed fluid therapy (GDFT) on pulmonary complications after thoracic surgery. Forest plots for pulmonary complications are shown with the pooled RR

**Fig. 4:**
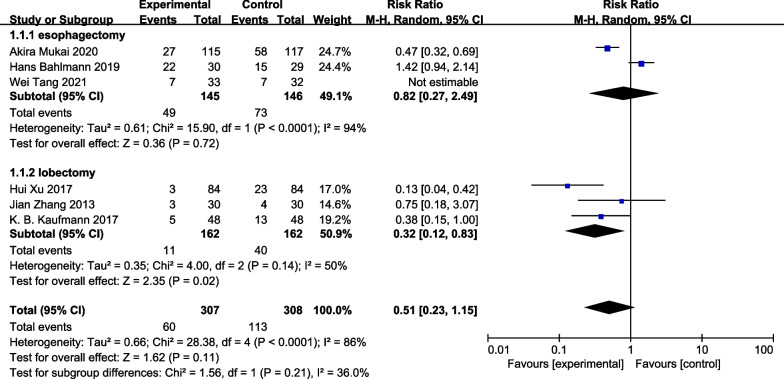
Forest plot of subgroup analyses about surgery types and the incidence of PPCs

All six included RCTs (680 patients) reported incidence of PPCs. Incidence of PPCs were 19.7% (67/340) in the GDFT group and 35.3% (120/340) in the control group, with no significant difference between these groups (RR 0.57, 95% CI 0.29–1.14; I^2^ = 82%, *P* = 0.11) (Fig. 3). As significant heterogeneity was observed, data were analyzed with a random-effects model. Subgroup analyses were conducted to explore the impact of GDFT on the incidence of PPCs in patients who underwent different surgery types. Subgroup analyses showed that GDFT decreased the incidence of PPCs in individuals who underwent lobectomy (RR0.32 95% CI [0.12, 0.83], *P* = 0.02; I^2^ = 50%; n = 3 [13, 14, 18]). (Fig. 4).

## Secondary outcomes

### Duration of hospitalization

**Fig. 5:**
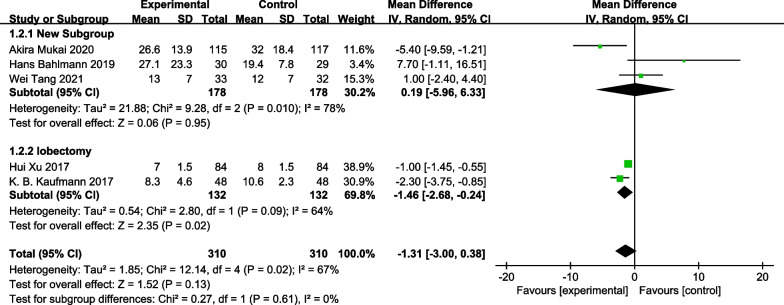
Forest plot for the duration of hospitalization. The sizes of squares for odds ratios reflect the weight of the trial in pooled analyses. Horizontal bars represent 95% confidence intervals

A total of five RCTs [13–15, 19, 20] provided sufficient data to assess the duration of hospitalization in study patients, revealing no significant difference between the control and GDFT groups [MD = − 1.31, 95% CI (− 3.00, 0.38), *P* = 0.13]. Subgroup analyses were conducted to explore the impact of GDFT on the hospitalization duration of patients who underwent different surgery types. Subgroup analyses showed that GDFT shortened the hospitalization in individuals underwent lobectomy. MD = − 1.46, 95% CI [− 2.68, − 0.24], *P* = 0.02; I^2^ = 64%; n = 2 [13, 14]). No decrease in hospitalization duration was evident for individuals undergoing esophagectomy (MD = 0.19, 95% CI [− 5.96, 6.33], *P* = 0.95; I^2^ = 78%; n = 3 [15, 19, 20] (Fig. 5).

### Postoperative renal dysfunction rates

**Fig. 6:**
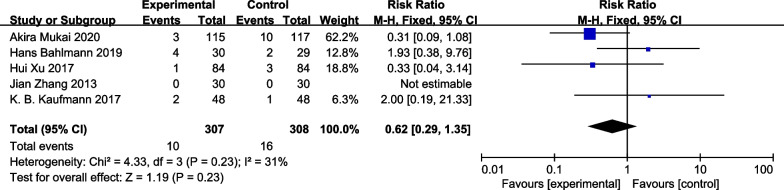
Forest plot for postoperative renal dysfunction rates

Five of the six studies assessed rates of renal dysfunction. No differences in these rates were observed between the GDFT and control groups when data were analyzed with a random-effects model [RR = 0.62, 95% CI [0.29, 1.35], *P* = 0.23, I^2^ = 31%] (Fig. 6).

### Postoperative cardiac complications rates

**Fig. 7:**
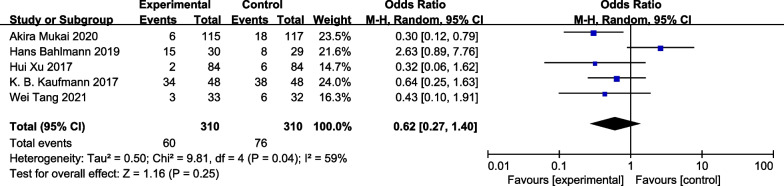
Forest plot for postoperative cardiac complications rates

Five studies [13–15, 19, 20] provided data sufficient to assess postoperative cardiac complication rates in analyzed patients. No differences in these rates were observed between the GDFT and control groups when data were analyzed with a random-effects model [RR = 0.62, 95% CI [0.27, 1.40], *P* = 0.25, I^2^ = 59%] (Fig. 7).

### Total intraoperative fluid input

**Fig. 8:**
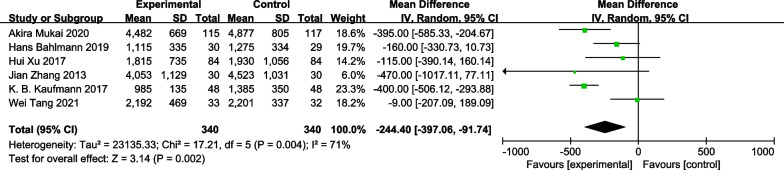
Meta-analysis and pooled weighted mean differences (WMDs) assessing the effects of GDFT on total intra-operative fluid input

Significant heterogeneity was detected among studies with respect to this endpoint, and as such, data were analyzed with a random-effects model. All five studies assessed total intraoperative fluid input, revealing a significantly lower fluid input in the GDFT group relative to the control group (MD =  − 244.40 ml [95% CI − 397.06, − 91.74], I^2^ = 71%, *P* = 0.002) (Fig. 8).

## Discussion

Six studies with 680 participants were included in the data synthesis. In our study, the incidence of PPCs in the control and GDFT groups was 35.3% and 19.7%, respectively. Although a descending trend was observed in the GDFT group, there was no statistically significant difference between the two groups. In addition, subgroup analysis did suggest that GDFT was associated with a decreased incidence of PPCs and duration of hospitalization for individuals undergoing lobectomy. However, the results are limited by the presence of substantial clinical and moderate statistical heterogeneity. Consequently, we graded the strength of evidence as moderate using the GRADE system [21]. This study is a comprehensive systematic analysis of RCT outcomes that use perioperative GDFT in individuals undergoing thoracic surgery.

The most common adverse events after thoracic lobectomy are pneumonia and atelectasis. Prior reports showed that GDFT could reduce rates of pneumonia, gastric tissue necrosis, and mediastinal abscess formation and decrease ICU hospitalization time to > 48 h in esophagectomy patients [22]. In one recent meta-analysis, the use of Goal-directed hemodynamic therapy (GDHT) was found to reduce the incidence of postoperative pulmonary infections and pulmonary edema in individuals undergoing general, abdominal, and cardiothoracic surgery [12]. Unlike these studies, in our systematic analysis, we failed to detect reductions in overall morbidity of PPCs in individuals undergoing thoracic surgery. Our results showed a trend toward GDFT being associated with a lower incidence of PPCs, but the limited sample size precluded this trend from reaching statistical significance. A larger sample size might have shown differences.

Recent evidence suggested that improper fluid administration in patients undergoing thoracic lobectomy could result in higher rates of acute lung or renal injury, potentially increasing the risk of respiratory and/or renal failure and mortality [7]. For patients undergoing one-lung mechanical ventilation, an imbalance between pulmonary ventilation and the blood flow ratio, elevated airway pressure, and excessive fluid administration could increase the risk of pulmonary complications such as acute respiratory distress syndrome (ARDS), which reportedly occurs at rates of 14.5–16% [23, 24]. Such excess intraoperative fluid infusion is thus closely associated with rates of postoperative mortality and other serious adverse events, including heart failure and pulmonary edema. Acute lung injury risk could be mitigated by avoiding excessive fluid loading following lobectomy. However, restrictive fluid infusion approaches could decrease renal perfusion and potentially increase mortality.[25–27].

Optimizing the intraoperative delivery of fluids is a key approach to improving the postoperative prognosis of thoracic surgery patients. GDFT is a personalized perioperative fluid management strategy wherein fluid administration targets are continuously adjusted based on key hemodynamic variables, including stroke volume, stroke volume variation, cardiac output, pulse pressure variation, and other factors, to optimize oxygen delivery and tissue perfusion [28].

GDFT aims to find the right balance between hypovolaemia and fluid overload and has been linked to a lower risk of postoperative adverse events in individuals undergoing high-risk operations [29]. Conducting GDFT based on objective, personalized metrics could prevent volume deficits while avoiding excessive fluid infusion. In this meta-analysis, we determined that GDFT decreased overall intraoperative fluid infusion. Although the GDFT group had a lower fluid infusion than the control group, the incidence of PPCs, postoperative myocardial injury, and renal insufficiency did not differ. One possible explanation is that SVV-based GDFT protocol has inherent limitations, especially in thoracic surgery. The usefulness of hemodynamic variables, such as SVV, is questioned in thoracoscopic surgery and one-lung ventilation when using small tidal volumes and CO_2_ artificial pneumothorax. However, that does not mean SVV has lost the usefulness as a predictor of fluid responsiveness in cardiothoracic surgery completely. In the past decade, more and more studies supported that SVV can be used in thoracoscopic one-lung ventilation and can better reflect the patient's fluid reactivity [30–32]. Due to small sample sizes, different mechanical ventilation settings, fluid reactivity monitoring methods, and observation time points, there was substantial heterogeneity across studies that resulted in conflicting published findings. In order to improve the predictability of SVV to fluid responsiveness, in our meta-analysis, the studies that used SVV-guided fluid management were setting the individualized SVV diagnostic threshold or combination cardiac index parameters. Overall, we cannot deny the value of SVV in guiding perioperative fluid management and reducing postoperative complications and mortality undergoing thoracic surgery.

Many different GDFT strategies have been implemented in clinical settings without consensus regarding the most appropriate monitoring strategy. SVV and transesophageal Doppler have both been widely used in this context in recent years [33–35]. Kaufmann et al. [14] found that relative to standard hemodynamic management strategies, GDFT guided by esophageal Doppler was linked to lower rates of PPCs and a shorter duration of hospitalization. Nevertheless, as this was the only study that used esophageal Doppler monitoring strategy, we were unable to perform appropriate subgroup analyses of these data.

In subgroup analyses, our results showed that GDFT was associated with shortened hospitalization and decreased incidence of PPCs among individuals undergoing lobectomy without any comparable decrease in esophagectomy patients. This suggests that GDFT might be more suitable for patients with lobectomy.

A recent systematic review and meta-analysis showed that GDFT during general anesthesia might decrease the duration of hospitalization, mortality, and several postoperative complications [36]. In addition, a previous study indicated that intraoperative goal-directed therapy might reduce major morbidity and mortality after transthoracic oesophagectomy [14]. In the present study, data of the postoperative mortality rates were counted in two of the six studies. The mortality rates were not conducted due to the insufficient number of studies included. The next step in our future study is to look at the mortality to determine whether the reduction in PPCs translates into clinical benefit, whether this be improved survival to discharge, improved quality of life.

There are a number of limitations to this meta-analysis that warrant consideration. First, the number of studies and the associated sample size were relatively small, resulting in the vulnerability of the results to Type II error. Second, only studies published in English were included in this analysis, thus potentially introducing a degree of selection bias. Third, only published data were eligible for inclusion in this analysis, and the absence of so-called “grey literature” evidence may have thus led to some degree of publication bias. Fourth, the included studies included a range of surgical approaches, hemodynamic monitoring strategies, objectives, and intervention timings, potentially resulting in relatively high clinical heterogeneity. Additionally, the utility of functional hemodynamic variables such as SVV when using small tidal volumes is limited, and it remains unclear whether these variables are relevant when the chest is open. In light of the above limitations, there are only 6 RCTs that are heterogeneous to make a conclusion. Thus, additional large-scale multi-center RCTs with a strict study design and a long follow-up period will be necessary to confirm the results of this study.

## Conclusion

The results of this meta-analysis indicate that GDFT was not reduced the postoperative incidence of pulmonary complications in individuals undergoing thoracic surgery. However, these results should be interpreted with caution considering the small number of contributing studies.

### Supplementary Information

**Additional file 1: **Prisma Checklist.

**Additional file 2**: Search details.

## Data Availability

All data generated or analysed during this study are included in this published article [and its supplementary information files].

## Abbreviations

GDFT: Goal-directed fluid therapy
RCTs: Controlled trials
RRs: Risk ratios
WMDs: Weighted mean differences
CIs: Confidence intervals
MAP: Mean arterial pressure
SVV: Stroke volume variability
SV: Stroke volume
ARDS: Acute respiratory distress syndrome
