# Supplementary material for: The impact of goal-directed fluid therapy on postoperative pulmonary complications in patients undergoing thoracic surgery: a systematic review and meta-analysis
Source: J Cardiothorac Surg. 2024 Feb 5;19:60. doi: 10.1186/s13019-024-02519-y (PMC10840200; doi:10.1186/s13019-024-02519-y)
Supplement: Supplementary file 2 — Additional file 2: Search details. [file 13019_2024_2519_MOESM2_ESM.doc]

**Sources PubMed**

**Search: ((((((((((((((("Thoracic Surgery"[Mesh]) OR (thoracic surgery[Title/Abstract])) OR ("Pneumonectomy"[Mesh])) OR (lung resection[Title/Abstract])) OR (lobectomy[Title/Abstract])) OR (Pneumonectomy[Title/Abstract])) OR ("Esophagectomy"[Mesh])) OR (esophagectomy[Title/Abstract])) OR (esophagectomies[Title/Abstract])) OR (oesophagectomies[Title/Abstract])) OR (oesophagectomy[Title/Abstract])) OR (One-Lung Ventilation[Title/Abstract])) OR (OLV[Title/Abstract])) OR ("One-Lung Ventilation"[Mesh])) OR (single-lung ventilation[Title/Abstract])) AND (((((((("Fluid Therapy"[Mesh]) OR ("Early Goal-Directed Therapy"[Mesh])) OR (GDFT[Title/Abstract])) OR (goal‐directed fluid therapy[Title/Abstract])) OR (fluid restriction[Title/Abstract])) OR (fluid optimization[Title/Abstract])) OR (fluid administration[Title/Abstract])) OR (fluid therapy[Title/Abstract]))**

**Sources Cochrane Library**

**ID Search Hits**

**#1 MeSH descriptor: [Thoracic Surgery] explode all trees 169**

**#2 (Thoracic Surgery):ti,ab,kw (Word variations have been searched) 6432**

**#3 MeSH descriptor: [Pneumonectomy] explode all trees 578**

**#4 (lung resection):ti,ab,kw (Word variations have been searched) 3783**

**#5 (lobectomy):ti,ab,kw (Word variations have been searched) 1553**

**#6 ("esophagectomy"):ti,ab,kw (Word variations have been searched) 1222**

**#7 (esophagectomies):ti,ab,kw (Word variations have been searched) 1222**

**#8 (oesophagectomies):ti,ab,kw (Word variations have been searched) 253**

**#9 (oesophagectomy):ti,ab,kw (Word variations have been searched) 254**

**#10 ("one-lung ventilation"):ti,ab,kw (Word variations have been searched) 712**

**#11 (OLV):ti,ab,kw (Word variations have been searched) 372**

**#12 MeSH descriptor: [One-Lung Ventilation] explode all trees 105**

**#13 (single-lung ventilation):ti,ab,kw (Word variations have been searched) 108**

**#14 MeSH descriptor: [Fluid Therapy] explode all trees 1745**

**#15 MeSH descriptor: [Early Goal-Directed Therapy] explode all trees 10**

**#16 (GDFT):ti,ab,kw (Word variations have been searched) 81**

**#17 (goal‐directed fluid therapy):ti,ab,kw (Word variations have been searched) 566**

**#18 (fluid restriction):ti,ab,kw (Word variations have been searched) 1643**

**#19 (fluid optimization):ti,ab,kw (Word variations have been searched) 1744**

**#20 (fluid administration):ti,ab,kw (Word variations have been searched) 9817**

**#21 (fluid therapy):ti,ab,kw (Word variations have been searched) 15119**

**#22 #1OR#2OR#3OR#4OR#5OR#6OR#7OR#8OR#9OR#10OR#11OR#12OR#13 11577**

**#23 #14OR#15OR#16OR#17OR#18OR#19OR#20OR#21 20277**

**#24 #22AND#23 297**

**Sources Web of Science**

**#1 TS=(Thoracic Surgery) OR TS=(Pneumonectomy) OR TS=(lobectomy) OR TS=(lung resection) OR TS=(Esophagectom*) OR TS=(oesophagectom*) OR TS=(One-Lung Ventilation) OR TS=(OLV) OR TS=(single-lung ventilation)**

**#2 TS=(Fluid Therapy) OR TS=(Goal-Directed Therapy) OR TS=(fluid restriction) OR TS=(fluid optimization) OR TS=(fluid administration)**

**#3 #1 AND #2**

**Sources Embase**

**Query ('lung resection'/exp OR 'thorax surgery'/exp OR 'one lung ventilation'/exp OR 'thoracic surgery':ab,ti OR 'lung resection':ab,ti OR lobectomy:ab,ti OR esophagectomy:ab,ti OR oesophagectomy:ab,ti OR olv:ab,ti OR 'single-lung ventilation'/exp OR 'single-lung ventilation' OR ('single lung' AND ('ventilation'/exp OR ventilation))) AND ('fluid therapy'/exp OR 'early goal-directed therapy'/exp OR gdft:ab,ti OR 'goal‐directed fluid therapy':ab,ti OR 'fluid restriction':ab,ti OR 'fluid optimization':ab,ti OR 'fluid administration':ab,ti)**
